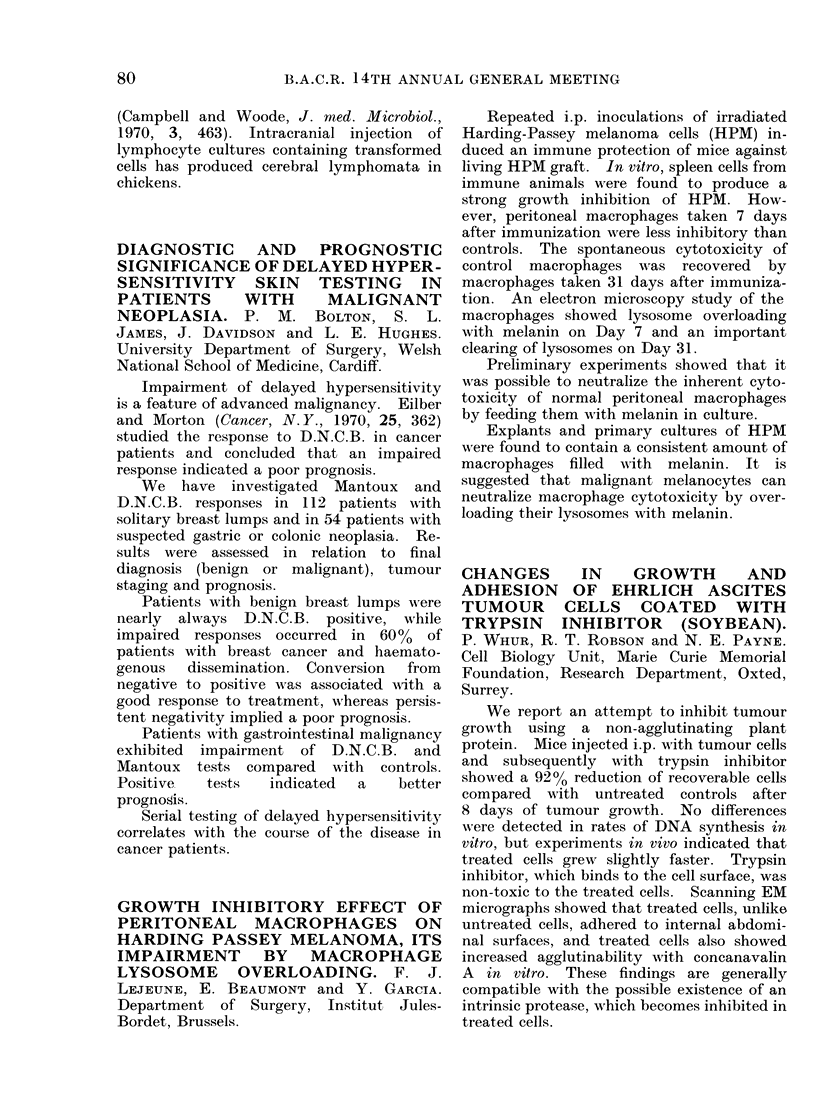# Changes in growth and adhesion of Ehrlich ascites tumour cells coated with trypsin inhibitor (soybean).

**DOI:** 10.1038/bjc.1973.88

**Published:** 1973-07

**Authors:** P. Whur, R. T. Robson, N. E. Payne


**CHANGES IN GROWTH AND ADHESION OF EHRLICH ASCITES TUMOUR CELLS COATED WITH TRYPSIN INHIBITOR (SOYBEAN).** P. Whur, R. T. Robson and N. E. Payne. Cell Biology Unit, Marie Curie Memorial Foundation, Research Department, Oxted, Surrey.

We report an attempt to inhibit tumour growth using a non-agglutinating plant protein. Mice injected i.p. with tumour cells and subsequently with trypsin inhibitor showed a 92% reduction of recoverable cells compared with untreated controls after 8 days of tumour growth. No differences were detected in rates of DNA synthesis *in vitro*, but experiments *in vivo* indicated that treated cells grew slightly faster. Trypsin inhibitor, which binds to the cell surface, was non-toxic to the treated cells. Scanning EM micrographs showed that treated cells, unlike untreated cells, adhered to internal abdominal surfaces, and treated cells also showed increased agglutinability with concanavalin A *in vitro*. These findings are generally compatible with the possible existence of an intrinsic protease, which becomes inhibited in treated cells.